# Patient satisfaction with the healthcare system: Assessing the impact of socio-economic and healthcare provision factors

**DOI:** 10.1186/s12913-016-1327-4

**Published:** 2016-03-15

**Authors:** Sofia Xesfingi, Athanassios Vozikis

**Affiliations:** Department of Economics, University of Piraeus, 18534 Piraeus, Greece

**Keywords:** Patient satisfaction, Healthcare provision, Public health, Private health, Healthcare quality

## Abstract

**Background:**

Patient satisfaction is an important measure of healthcare quality as it offers information on the provider’s success at meeting clients’ expectations and is a key determinant of patients’ perspective behavioral intention. The aim of this paper is first to assess the degree of patient satisfaction, and second, to study the relationship between patient satisfaction of healthcare system and a set of socio-economic and healthcare provision indicators.

**Methods:**

This empirical analysis covers 31 countries for the years 2007, 2008, 2009 and 2012. The dependent variable, the satisfaction index, is defined as the patient satisfaction of their country’s health system. We first construct an index of patient satisfaction and then, at a second stage, this index is related to socio-economic and healthcare provision variables.

**Results:**

Our findings support that there is a strong positive association between patient satisfaction level and healthcare provision indicators, such as nurses and physicians per 100,000 habitants, with the latter being the most important contributor, and a negative association between patient satisfaction level and number of hospital beds. Among the socio-economic variables, public health expenditures greatly shape and positive relate to patient satisfaction, while private spending on health relates negatively. Finally, the elder a patient is, the more satisfied with a country’s healthcare system appears to be.

**Conclusions:**

We conclude that there is a strong positive association between patient satisfaction and public health expenditures, number of physicians and nurses, and the age of the patient, while there is a negative evidence for private health spending and number of hospital beds.

## Background

Quality of care is a dominant concept in quality assurance and quality improvement programs in the healthcare sector. The importance of quality in the healthcare sector has been recognized, but it has been accelerated over the last decade through the development of quality insurance, quality improvement programs and patients’ agendas [1]. While quality of care, rather than price, is the main concern in healthcare [2], the service provider’s technical competence, as well as the immediate results from many treatments, is very difficult for a patient to evaluate [3].

It has been proposed that we can measure the quality of healthcare by observing its structure, its processes and its outcomes [4]. Whereas the aims of effectiveness and safety of healthcare are nearly universal, societies and cultures around the world differ in how much they emphasize the additional aims of patient-centeredness, timeliness, efficiency and equity. Healthcare measures –including process measures– are developed for varied audiences who may wish to use them for healthcare purchasing, utilization, or performance improvement [5]. For all these purposes it is imperative that are meaningful, scientifically sound, generalizable, and interpretable [6].

Patient satisfaction is an important measure of healthcare quality as it offers information on the provider’s success at meeting the expectations of most relevance to the client [7] and a key determinant of patients’ perspective behavioral intention [8]. Patient satisfaction is correlated with important outcomes, such as superior compliance, decreased utilization of medical services, less malpractice litigation and better prognosis [7]. The absence of a solid conceptual basis and consistent measurement tool for consumer satisfaction has led, over the past ten years, to a proliferation of surveys that focus exclusively on patient experience, i.e., aspects of the care experience such as waiting times, the quality of basic amenities, and communication with healthcare providers, all of which help identify tangible priorities for quality improvement [9]. Some researchers have suggested that defining quality improvement from patients’ perspective provides better value for their money with improved safety, accessibility, equity, and comprehensiveness of care, while from a provider’s point of view, quality improvement may be more efficient, providing more effective services to a greater number of consumers with a reasonable level of satisfaction, with the latter being enough for customer retention [10].

A handful of studies have attempted to relate patient’s health status to factors such as the performance of healthcare system [9] or other demographic and economic factors [11–13]. For a comprehensive review on patient satisfaction, see Pascoe [14] and Naidu [15].

More specifically, Bleich et al. [9] find that with respect to patient satisfaction and for 21 EU countries for the year 2003, about a quarter of the variation is attributed to healthcare system itself and to patient expectations, health status, type of care and immunization coverage. Furthermore, the study of Mummalaneni and Gopalakrishna [11] examines socio-demographic factors such as age, gender, occupation, employment status, education and income and reveals that income is the only socio-demographic factor found to have an influence on patient satisfaction. In addition, Gordo [12] examines data from the German Socio-Economic Panel and finds a strong association between long-term unemployment and patient satisfaction, while a weak association is documented for the short-term unemployment and patient satisfaction depending on the gender. Lastly, the study of Popescu et al. [13] investigates health status in relation to expenditures on health along with healthcare provisions (hospital beds and physicians per person) and finds a strong relationship between reporting a good or bad health status and health expenditures and provisions. A relevant study, that of Zhao et al. [16], examines instead the willingness to pay (WTP) per Quality-Adjusted Life Year (QALY) for a sample of chronic prostates patients. The WTP is associated with demographic factors of patients such as age, gender, education, marital status and with economic factors such as employment and level of income.

The purpose of this paper is first, to map the degree of the patient satisfaction in relevance with the healthcare system of their country during the years 2007, 2008, 2009 and 2012 in a panel of 31 countries using a satisfaction index, and second, to assess the impact of socio-economic and healthcare provision factors on the degree of patient satisfaction.

The contribution of this study is twofold. First, the hospital performance is transformed into a satisfaction index based on the patient’s perceptions about their country healthcare system. The latter, consists the first attempt in the literature. Second, the degree of patient satisfaction is examined along with a set of socio-economic and healthcare provision indicators. This is the first time in the literature as the majority of relevant studies explore only some indicators and for a limited number of countries and years.

The remaining of this paper is organized as follows: Section 2 presents our framework of analysis, data and model. Section 3 presents and discusses our findings. Finally, Section 4 concludes.

## Methods

### Data

Quality of care from the patient’s perspective and patient satisfaction are two major multidimensional concepts that are used several times interchangeably [1]. Within this framework, every consumer or citizen may be a potential patient. This empirical analysis covers 31 countries: 28 EU Member States, Iceland, Norway, and Switzerland. The dependent variable, the satisfaction index, is defined as the patient satisfaction with respect to the country’s healthcare system, for the years 2007, 2008, 2009 and 2012. Information for the years 2010 and 2011 was not available. Furthermore, some of the countries did not provide data at the time of our research, especially with respect to healthcare provision factors, and, more particularly, for the number of nurses corresponding per 1000 habitants. Therefore, our final data set consists of 88 observations (instead of 124 = 31*4) as, for robustness purposes, we exclude the countries with limited data.

For the construction of the satisfaction index, data for the corresponding years were used from the Euro Health Consumer Powerhouse, and particularly, from the Euro Health Consumer Indexes (EHCI), where the performance of a country’s health system is evaluated through personal interviews and an active feedback from national healthcare agencies and institutions [17]. The built of EHCI is based on several indicators grouped in seven sub-disciplines, namely “Patient Rights and Information (PRI)”, “Accessibility (ACC)”, “Outcomes (OUT)”, “Range (RAN)”, “Pharmaceuticals (PHA)”, “Prevention (PRE)” and “E-Health (E-HEA)”. The performance of the respective national healthcare system is graded on a three-grade scale for each indicator, where the grades have the rather obvious meaning of “Good” = 3 points, “So-so” = 2 points and “Not so good” = 1 point. For each of the sub-disciplines, the country score is calculated as a percentage of the maximum possible, and then multiplied by weight coefficients since certain indicators are being more important than others.

For example, the sub-discipline “ACC” for the year 2012 consists of five indicators. Therefore, the maximum possible score is 5*3 = 15. The weight coefficient for this sub-discipline, along with the suggestions of expert panels and experience from a number of patient survey studies, is 250. Therefore, the points taken for every “Good” evaluation is 50. Consequently, the maximum score attainable for a national healthcare system, adding up all sub-disciplines scores after multiplying them with the weight coefficients is 1000 and the lowest possible score is 333.

We used the sub-disciplines’ total scores for each country to construct the Satisfaction Index. A country’s satisfaction index is a dummy and takes the value of 1 if its satisfaction index value is above the sample average; otherwise is 0.

**Fig. 1:**
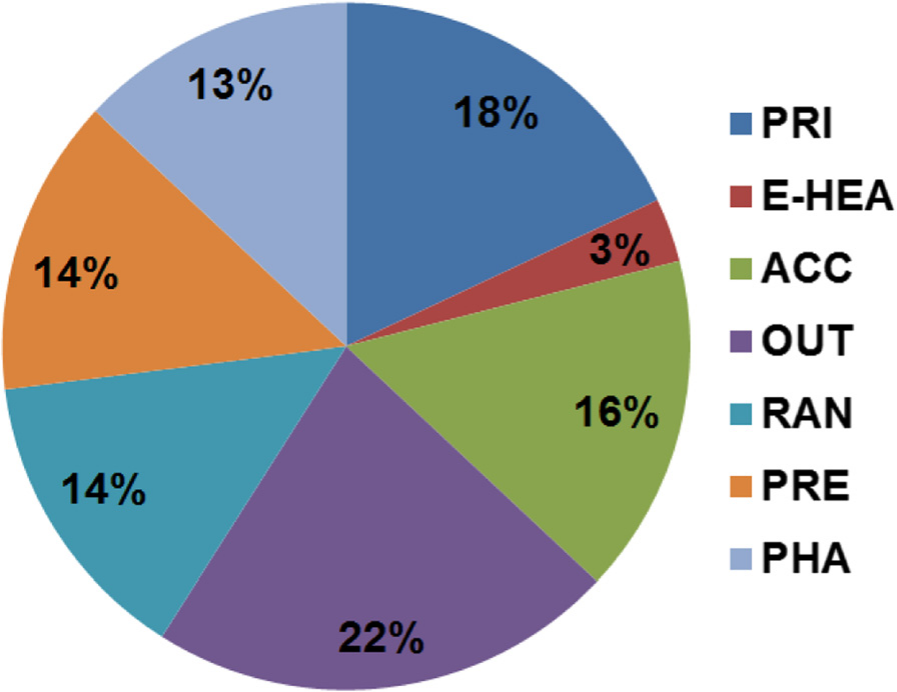
Share of the sub-disciplines of the satisfaction index

Figure 1 shows the distribution of each one of seven sub-disciplines of the satisfaction index.

A number of (macro)economic indicators were employed, such as Gross Domestic Product (GDP) per capita (measured in constant 2005 US$), Health Expenditures as a percentage of GDP, Public Health Expenditures as a percentage of GDP, Private Health Expenditures as a percentage of GDP, Unemployment rate as percentage of total labor force, and Population Aging as a percentage of the total population above the age of 65 years, obtained from World Bank [18]. Furthermore, we also include some healthcare provision indicators, namely Number of Physicians per 100,000 habitants, Number of Nurses per 100,000 habitants and Number of Hospital Beds per 100,000 habitants, from Eurostat [19].

Table 1 presents the summary statistics of all variables.

**Table 1:** Summary statistics of socio-economic and healthcare provision indicators

Variable	Mean	SD	Min	Max
Satisfaction	0.476	0.502	0	1
GDP per capita	27,588.72	19,801.13	4,339.468	79,295.53
Dummy	0.427	0.498	0	1
Health Expenditures (% GDP)	8.381	1.814	5.101	11.915
Public Health Expenditures (%)	6.213	1.679	2.578	9.756
Private Health Expenditures (%)	2.138	0.793	0.928	4.329
Unemployment (% labor force)	7.322	3.826	2.500	25.000
Population Aging (% total)	16.265	1.896	11.008	21.101
Physicians/100,000 habitants	323.635	63.460	212.128	482.381
Nurses/100,000 habitants	856.691	377.936	398.000	1,660.356
Hospital Beds/100,000 habitants	93.117	148.576	1.059	677.799

As Table 1 shows, countries in our sample spend about 8.4 % of their GDP on health. Specifically, public health expenditures are three times larger than private ones. Moreover, it appears that per 1000 habitants (i.e. patients), on average, correspond almost 3 physicians, 8 nurses, and 1 hospital bed (323.635/100 ≈ 3, 856.691/100 ≈ 8, and 93.117/100 ≈ 1).

### Model

The likelihood of a certain patient being satisfied of a country’s healthcare system can be described by a logit model defined as follows:

$$ \mathrm{Prob}\ \left(Y = 1\Big|{X}_{\mathrm{i}}\right) = \mathrm{F}\left({X}_{\mathrm{i}}\beta \right), $$

where the endogenous variable *Y* is the degree of patient’s satisfaction and takes the value 1, if the patient is satisfied with his/her country’s healthcare system, and 0 otherwise; *F* is the standard logistic cumulative distribution function and *Χ*_i_ is a set of covariates. The model is defined as:

$$ \begin{array}{l}{Y}_i={\beta}_0+{\beta}_1 GDPcapit{a}_{\mathrm{i}}+{\beta}_2 HealthExpenditure{s}_{\mathrm{i}}+\\ {}{\beta}_3 Unemploymen{t}_{\mathrm{i}}+{\beta}_4 PopulationAgin{g}_{\mathrm{i}}+\\ {}{\beta}_5 Physician{s}_{\mathrm{i}}+{\beta}_6 Nurse{s}_{\mathrm{i}}+{\beta}_7 HospitalBed{s}_{\mathrm{i}}+{\varepsilon}_{\mathrm{i}},{\varepsilon}_{\mathrm{i}}\sim \mathrm{Logistic}\left(0,1\right)\end{array} $$

where *GDPcapita* is gross domestic product (GDP) per capita, *HealthExpenditures* is public and private expenditures on health (%GDP), *Unemployment* is the unemployment rate, *PopulationAging* is the people above the age of 65 years old (%total population), *Physicians* is the number of physicians per 100,000 habitants, *Nurses* is the number of nurses per 100,000 habitants and *HospitalBeds* is the number of hospital beds per 100,000 habitants. The first four variables capture socio-economic conditions, whereas the remaining three proxy healthcare provision.

## Results

Table 2 presents the odds ratios for all specifications. The odd ratios can be interpreted as follows: if the odd ratios >1, then the probability of a patient being satisfied, i.e. *Y*_it_ = 1, increases, while decreases if odd ratios <1. Column (1) presents estimates of the baseline model, where health expenditures are aggregated into public and private spending. Column (2) splits the health expenditures into two categories, public and private health expenditures. For robustness purposes, columns (3) and (4), re-estimate specifications (1) and (2), but this time countries are classified as “high-income” and “low-income”. In doing so, a new variable, *Dummy*, is defined as follows: if a country’s GDP per capita is above sample average, then *Dummy* is one; otherwise is zero.

**Table 2:** Logit estimates (odds ratio) of different model specifications (dependent variable: *patient satisfaction*)

Variables	(1)	(2)	(3)	(4)
*GDPcapita*	1.00005	1.00004		
	(0.00005)	(0.0001)		
*Dummy (for income level)*			4.909	1.681
			(7.797)	(4.981)
*HealthExpenditures*	1.384		1.456	
	(0.619)		(0.613)	
*PublicExpenditures*		35.184^a^		34.797^a^
		(59.060)		(58.427)
*PrivateExpenditures*		0.013^b^		0.011^b^
		(0.031)		(0.027)
*Unemployment*	0.939	0.801	0.963	0.826
	(0.107)	(0.206)	(0.113)	(0.2)
*PopulationAging*	4.403^a^	89.652^a^	4.021^a^	85.65^a^
	(2.722)	(163.124)	(2.519)	(154.497)
*Physicians*	1.018^b^	1.069^a^	1.017^b^	1.066^b^
	(0.011)	(0.036)	(0.106)	(0.036)
*Nurses*	1.012^a^	1.026^b^	1.013^a^	1.028^a^
	(0.005)	(0.138)	(0.005)	(0.014)
*HospitalBeds*	0.987^b^	0.957^a^	0.986^a^	0.957^a^
	(0.007)	(0.018)	(0.007)	(0.018)
Observations	85	85	85	85
Likelihood Ratio (X^2^)	78.62	99.05	78.38	98.89
Pseudo-R^2^	0.667	0.841	0.665	0.839

Note: Numbers in parentheses are standard errors; (^a^), (^b^) indicate significance at 5 %, and 10 %, respectively

As Table 2 demonstrates, in specification (1), where one does not account for different type of health expenditures, i.e. public vs. private, the logit estimates are consistent with the theory and carry the right sign. Among the socio-economic variables, *GDPcapita, HealthExpenditures*, *Unemployment* and *PopulationAging*, only the latter appears to be statistically significant. More specifically, if population aging increases, the probability of a patient being satisfied increases by 340.3 % [(4.403-1)*100]. With respect to the healthcare provision indicators, all of them seem to be statistically significant. The number of physicians and the number of nurses associate positively with the patient satisfaction level (an increase in those indicators leads to an increase of the satisfaction level by 1.8 and 1.2 %, respectively) while the number of hospital beds associates negatively with patient’s satisfaction (an increase in number of hospital beds leads to a decrease of the patient satisfaction level by 1.3 %).

In column (2), once aggregated health expenditures are decomposed into public and private, findings appear somewhat different. Particularly, public health spending appears to be positively and statistically associated with patient satisfaction, that is, if public health expenditures increase, the probability of a patient being satisfied increases tremendously about 3500 times.

In order to capture the income differences across countries, the *Dummy* variable is introduced in the model in columns (3) and (4). The estimates of the baseline model still carry the right sign while the statistical significance pertains. Independently of a country’s income level, we find that the same set of variables associates in shaping patient satisfaction degree.

With respect to the overall performance of our specifications, correlations between *patient satisfaction* (*Y*_it_) and *predicted patient satisfaction* (*Ŷ*_it_) range from 84.5 to 92 % (at 5 % level of significance), indicating that the fitness of our specifications is satisfactory. The likelihood ratios from the diagnostics (bottom of Table 2), further confirm the goodness of the fit of our model.

## Discussion

Many studies have analyzed the relationship between GDP per capita and the health spending. These studies led to the extremely robust conclusion that even after statistical control for many other factors, the effect of GDP per capita (income) on expenditure is clearly positive and significant [20]. Public health expenditures play an important role for the patient satisfaction. Strong primary care has on better population health, fewer health disparities and lower rates of unnecessary hospitalizations [21]. Some countries are wealthy enough and they can afford to gear their governance, healthcare workforce, and funding arrangements towards expensive specialized care to satisfy public expectations [22].

If a patient is the habitant of a high-income country, his/her probability of being satisfied with the country’s health system is about 3400 times higher compared to a patient satisfaction level from a low-income country. This dramatic difference between “high-income” vs. “low-income” countries reflects the different perceptions existing among patients from different countries, implying that patients who reside in wealthier countries are more satisfied in general with the healthcare system compared to patients from less wealthy economies. It seems that wealthier countries are able to keep their patients more satisfied than poor ones, as expected.

The public spending on health has a large impact on patient satisfaction simply because health services are perceived to be provided free of charge by the state. The latter is more important for countries which are less wealthy. The important role of public health spending is also documented in numerous studies [23, 22]. In contrast, private health spending appears to be negatively correlated with patient satisfaction as an increase of private health expenditures decreases patient satisfaction by 98.7 %. The negative relation between private health spending and patient satisfaction seems reasonable if one takes into consideration that citizens of all countries, although contribute to public health expenditures through taxation, they pay out of their pockets to receive (better) private healthcare when public healthcare fails. This is also consistent with other studies findings [24].

According to Kotzian [23], the patient satisfaction with the healthcare system might be influenced by other economic factors and properties of the healthcare system. As pointed out in the same article, the healthcare system might work well, but the distribution of the financial burden for its financing might be considered unfair by the patients. For example, as habitants of each country are getting older, we expect them to spend more money for their health status. Therefore, it seems natural to conclude that a nation’s per capita health spending will rise significantly as the average age of its population rises and that cross-national variations in health spending per capita are driven significantly by cross-national variations in the percentage of the population that is age sixty-five and older [24]. This is in line with other studies showing that elderly patients are more likely to express satisfaction with their healthcare than other sections of the patient population [25].

When it comes to healthcare provision, the literature finds that patient-to-nurse workloads were significantly associated with patients’ ratings and recommendation of the hospital to others, and therefore with their satisfaction when discharged [26]. Furthermore, Kotzian [23] suggested that a relatively low level of physicians per capita indicates a relative shortage of medical staff, and this might lower the satisfaction in the sense that there are not enough personnel to deliver beyond-health outputs. In the study of Ghose and Adhish [27], it was observed that patient satisfaction was greatly influenced by timing of admission, medical research and development, pharmacy, pantry services, nursing care and doctor’s care. More specifically, a very high percentage of the patients were satisfied with the physician services like availability of the doctor, doctor’s care and the treatment given by them.

All healthcare provision indicators seem to be statistically significant, but their relationships with patient satisfaction level do not carry the same sign for all of them. Particularly, the positive associations between the number of physicians and the satisfaction level, as well as the number of nurses and the satisfaction level, have also been documented to have similar effects in other studies. These findings are consistent with the studies of Kutney-Lee et al. [26] and Kotzian [23]. However, this increase of doctors and/or nurses could lead to higher public expenses and in countries with high debt/deficit could be challenging. In addition, if the number of hospital beds increases, the probability of a patient being satisfied with the healthcare system decreases by 1.3 %. This finding may reflect the unsolved issue of overcapacity which is documented in several studies, such as Kosnik [28] and Fidler et al. [29].

Since there is the first time to our knowledge that the healthcare systems’ performance is transformed into patient satisfaction, it is worth to further evaluate the construction of the satisfaction index. There was no methodology to base upon and, in addition, there were missing data for some of the countries (for the variables or for the years). Possible changes or errors with respect to the way data were collected could influence the satisfaction index and, in consequence, the results. Our methodology though, for the construction of the satisfaction index, is based first on the sample average and second on the ranking of each country with respect to the aforementioned average. We try with alternative indices constructed with higher deviations with respect to the proposed one. Results do not change significantly. Rankings of each country do not change dramatically across the years, so we may assume that even with the addition of some data, a country would probably stay above or under the sample average. If one takes into consideration that all finding refer to the sum of all countries in question, it is quite difficult to interpret since countries are grouped in different categories according to their national health care system, and face different difficulties with respect to their public spending or their health care provision indicators.

Finally, there might be several confounding factors that have contributed to these findings. Although it has been demonstrated that educational level plays an important role in shaping patient satisfaction, the data did not provided this information. Therefore, further research could focus on a country-level analysis, taking into consideration omitted factors, and evaluate the use of the questionnaire as well as the possible ceiling effect.

Overall, a key factor of patient satisfaction seems to be the responsiveness of the national healthcare system and the strategic changes’ implementation. It is a general belief that the relatively richer countries, and with full-coverage healthcare systems, are the best performers but the true problem lies on communicating the considerable improvements to the wide public.

## Conclusions

This paper studied the relationship between patient satisfaction of a country’s healthcare system and a set of socio-economic and healthcare provision indicators.

Based on 31 countries and four years, our findings document the significant role of healthcare provision indicators such as the number of physicians and nurses provided in the healthcare system and support that there is a strong association between patient satisfaction level and number of hospital beds, nurses and physicians per 100,000 habitants, with the latter being the most important contributor. Among the socio-economic variables, public spending on health plays prominent role on patient satisfaction as greatly shapes and positive relates to patient satisfaction, while private spending on health related negatively. Finally, the elder a patient is, the more satisfied with a country’s healthcare system appears to be, exhibiting higher satisfaction from countries’ healthcare system.

A policy implication of our findings is that the role of government on health spending is highly important for patient satisfaction with respect to a healthcare system performance. Future research should control also for the type and quality of public as well spending in health.

## Abbreviations

ACC: Accessibility
EHCI: Euro Health Consumer Index
E-HEA: E-Health
EU: European Union
GDP: Gross Domestic Product
OUT: Outcomes
PHA: Pharmaceuticals
PRE: Prevention
PRI: Patient Rights and Information
QALY: Quality-Adjusted Life Year
RAN: Range
WTP: Willingness to pay
